# The trajectories of bioelectrical impedance analysis-derived raw variables (phase angle and impedance ratio) in healthy Italian children and adolescents: a retrospective observational study

**DOI:** 10.1007/s00431-026-06748-2

**Published:** 2026-01-24

**Authors:** Giada Ballarin, Dario Bruzzese, Paola Alicante, Olivia Di Vincenzo, Giuliana Valerio, Luca Scalfi

**Affiliations:** 1 Department of Medical, Movement and Wellbeing Sciences, University of Napoli “Parthenope”, 80133 Naples, Italy; 2 Department of Public Health, “Federico II” University, 80131 Naples, Italy; 3 Department of Experimental Medicine, “Sapienza” University, 00161 Rome, Italy

**Keywords:** Phase angle, Impedance index, Biolelectrical impedance analysis, Children, Adolescents

## Abstract

There is a growing interest for bioelectrical impedance analysis (BIA)-derived raw variables such as phase angle (PhA) and impedance-Z ratio, since they are considered proxy indicators of body cell mass and muscle structure. So far, limited information is available on the trajectories of PhA and IR during the first two decades of life. Anthropometry was measured with standardized procedures. For the whole body, PhA was measured at 50 kHz, while IR was calculated as the ratio of Z at 250 kHz/Z to that 5 kHz. Fat-free mass (FFM) and percentage body fat (%BF) were estimated by BIA. Segmented linear regression was applied to assess whether the relationships between PhA or IR and age showed discontinuities with time. Healthy children and adolescents were enrolled (302 boys and 278 girls aged 6–16 yrs). Independent predictors of PhA were body weight, height, FFM and %BF in boys and age, body weight, height and FFM in girls. Similar findings were observed for IR. PhA increased similarly in both sexes from 6 to 11 yrs. In the second decade the slope was ten times steeper in boys and three times in girls, with a breakpoint around 12 yrs in both sexes. A breakpoint was also detected for IR which was significant in boys only. This study provides a comprehensive description of the trajectories of PhA and IR in healthy children and adolescents. Changes of both variables were associated with age, with breakpoints observed around 11–12 yrs of age, followed by more marked variations with time.

**Table:** 

**What is known:** • *In children and adolescents, BIA-derived raw variables, such as phase angle and impedance ratio, have been related to muscle strength and cardiorespiratory fitness and were considered as a marker of outcome in hospitalized paediatric patients.* • *A limited number of papers described the trajectory of these variables in paediatric population. *
**What is new:** • *There are very clear age-related differences in BIA-derived variables over the first two decades of life, with a variability related to variation in fat-free mass.* • *For both variables, breakpoints were observed particularly in boys around 12 yrs of age.*

**Supplementary Information:**

The online version contains supplementary material available at 10.1007/s00431-026-06748-2.

## Introduction

The evaluation of body composition plays a key role in assessing nutritional status (both in research and clinical settings) and represents a dimension of health-related physical fitness across all stages of human life [1–3]. As for the first two decades of life, a greater percentage of body fat (%BF) and lower fat-free mass (FFM) have been found to be associated with insulin resistance, impaired glucose tolerance and metabolic syndrome [1, 4]. Of note, an excess of body fat in the first part of life is associated with a high risk of being obese in the adulthood [5]. On the other hand, FFM is positively related to health-related physical fitness [6], even in children with obesity [7].

It is indeed well known that substantial changes in body composition occur during the first two decades of life along with hormonal variations. Body weight, FFM, fat mass (FM), and bone mineral content increase in absolute terms [1, 8, 9], while sexual dimorphism begins to emerge with puberty [8, 10, 11], when a more marked increase in %BF is observed in females compared to males [7, 8].

Variables that are directly measured (e.g., raw) by bioelectrical impedance analysis (BIA) can provide further insight into body composition: phase angle (PhA) and the impedance ratio (IR) are supposed to be proxy indicators of body cell mass (BCM), of the ratio of intracellular water to total body water (ICW/TBW ratio) and overall cellular integrity, in both health and disease [2, 12].

In children and adolescents PhA has been linked to muscle strength and cardiorespiratory fitness [13–18] and also emerged as a predictor of outcome in hospitalized paediatric patients [18]. A limited number of papers has shown that PhA is higher in adolescents than children [9, 19–21], and in males compared to females post-puberty, consistent with the expected increase in BCM and ICW/TBW ratio [22]. Given that PhA increases over the first two decades of life [9, 20, 21, 23], so far these variations have not been explored in a systematic and specific way (i.e., illustrated only through figures) or not investigated at all, and the exact trajectory of these changes remains poorly defined.

IR has been even less studied in the first decades of life, being described as a proxy of illness severity in paediatric patients after heart surgery or related to inflammatory bowel disease [24, 25]. Indeed, no information is so far available on its changes in healthy children and adolescents.

Even if the use of PhA and IR is free of intrinsic errors due to the use of predictive equations for body composition, their interpretation remains challenging in both research and clinical settings, and more should be known about their physiological variations in the first two decades of life. Against this background our study was aimed to: (1) compare sex- and age-related differences of PhA and IR in healthy children and adolescents; (2) identify the predictors of PhA and IR; (3) analyze the trajectories of PhA and IR separately in the two sexes.

## Methods

### Participants

This is a retrospective observational study based on historical data collected from September 2022 to March 2023 and gathered from previous studies aimed at evaluating the association between anthropometric measurements, body composition as assessed by BIA and health-related physical fitness in a community sample of children and adolescents. These studies shared the same protocols and design. Specifically, individuals had been recruited using a two-stage cluster-sampling design. From a list of all schools in the district, three schools (one primary, one middle, and one high school) were randomly selected (stage 1). From each selected school, 10 classes were randomly chosen (stage 2). All students enrolled in these selected schools were then invited to participate by their class teachers.

Parents received a questionnaire together with the informed consent form, in which they provided information regarding their family origins and relevant aspects of their children's medical history, including the presence of metabolic disorders, recent hospitalizations or acute illnesses, ongoing chronic medication use, or implanted medical devices. Inclusion criteria were: (1) Italian or Southern European origin, defined as having all four grandparents and both parents born in Italy or Southern Europe (students with mixed origins were excluded to ensure sample homogeneity; ancestry was self-reported); (2) both sexes; and (3) provision of written informed consent. Exclusion criteria included: (1) the presence of metabolic disorders or other clinical conditions likely to affect body composition (as identified during interviews); (2) recent hospitalization, acute illness, or chronic medication use; and (3) the presence of implanted medical devices. A total of 629 students were eligible for recruitment. Five were excluded due to metabolic disorders or recent hospitalization, while 44 missed the assessment due to lack of parents’ consent or absence on the day of the examination. A final sample of 580 healthy children and adolescents was obtained: 278 girls (11.1 ± 3.1 years, range 5–17) and 302 boys (11.4 ± 3.1 years, range 5–17), yielding a 92.2% participation rate. For statistical purposes, participants were also subdivided into two subgroups: children (first decade of life, 5–10 yrs) and adolescents (second decade of life, 11–17 yrs). No missing data were present in the final dataset.

Written informed consent was obtained from all participants and their parents/guardians of minors. This study was performed in line with the principles of the Declaration of Helsinki. The study protocol was approved by the Ethics Committee of the *Federico II* University of Naples (protocol number 42/17).

### Anthropometric measurements

All measurements were collected in school facilities. Body weight and height were measured by a trained investigator, with participants wearing light clothes and no shoes, to the nearest 0.1 kg and 0.5 cm, respectively (SECA 813 portable scale and SECA 220 portable stadiometer, Hamburg—Germany). Body mass index (BMI) was calculated as weight in kg divided by the square of height in m (m^2^). Weight Z-score, height Z-score and body mass index Z-score (BMI Z-score) were calculated according to the World Health Organization growth reference for school-age children and adolescents [26]. Overweight was defined as a BMI Z-score value ≥ 1 while obesity and severe obesity were defined as BMI Z-score values ≥ 2 and ≥ 3, respectively.

### Bioelectrical impedance analysis

All BIA measurements were performed between 8:30 and 11:30 a.m., with a single measurement for each participant. Applying multifrequency BIA, Z was measured at frequencies 5–10–50–100–250 kHz and PhA at 50 kHz, under standardized conditions (ambient temperature between 23–25 °C, fasting > 3 h, empty bladder, supine position for at least 10 min), using a Human Im Touch analyser (© DS Medica S.r.l., Milan, Italy). After cleaning the skin, subjects were asked to be supine with their legs and arms slightly abducted to prevent contact between body segments. Measuring electrodes were placed on the anterior surface of the wrist and ankle, while injecting electrodes were placed on the dorsal surface of the hand and foot. FIAB PG500 electrodes were used as suggested by the manufacturer.

The following BIA-derived raw variables (mean values for right and left body sides) were considered: 1) PhA at 50 kHz; 2) IR calculated as Z at 250 kHz/Z at 5 kHz. As for body composition, FFM was estimated from Z at 50 kHz using a BIA equation developed in children aged 4–18 yrs [27]. FM was calculated as the difference between body weight and FFM, while %BF as the ratio of FM to body weight.

### Statistical analysis

Results are presented as mean ± standard deviation. Statistical significance was pre-determined as p < 0.05 and p < 0.001. All statistical analyses were performed using the Statistical Package for Social Sciences (SPSS Inc, Chicago, IL, USA) version 28 or R software (Core Team, 2021; R Foundation for Statistical Computing, Vienna, Austria. URL https://www.R-project.org/).

An a priori minimum sample size per sex of 287 participants was necessary to have adequate power (≥ 0.80) to detect a correlation of r = 0.20 as statistically significant (p < 0.01).

The Kolmorgov-Smirnov test was used to assess normality of distribution for each continuous variable. One-way analysis of variance (ANOVA) and the Tukey test were applied for comparing groups and the general linear model (GLM) to assess the effect of sex and age group on single dependent variable. Spearman’s rho was computed to examine correlations between variables. Partial correlation and multiple regression analysis were utilized to identify predictors of a given dependent variable. Residual analysis was performed to assess the reliability of linear regression models. Effect sizes were determined by calculating Cohen’s *d* on normally distributed variables [28]. Segmented linear regression was applied to assess potential discontinuities in the relationship of PhA or IR with age. This approach allows to automatically detect the presence and, if the case, the position of these breakpoints in an unsupervised manner [29].

## Results

The characteristics of the whole sample, stratified by sex and age groups (6–10 yrs and 11–17 yrs), are shown in Table 1. A significant difference in weight Z-score and BMI Z-score was observed between sexes in participants aged ≤ 10 yrs. In the second decade of life, height, weight and FFM were higher, while FM and %BF were lower in male compared to female participants (Table 1).

**Table 1:** Baseline characteristics of children and adolescents participating in the study

*N* = 580	Boys		Girls	
	5–10 yrs (*n* = 113)	11–17 yrs (*n* = 189)	5–10 yrs (*n* = 115)	11–17 yrs (*n* = 163)
Age (age)	8.1± 1.2	13.4± 2.1	8.1± 1.1	13.3± 2.1
Weight (kg)	33.9± 9.7c	56.7± 15.9ac	32.8± 11.1c	53.3± 13.5ac
Height (cm)	131.0± 7.7c	159.8± 13.1bc	129.9± 9.5c	155.3 ± 8.4bc
BMI Z-score	0.62± 1.10bd	0.28 ± 1.19d	0.36± 1.24b	0.34± 1.20
Weight Z-score	1.51± 1.45ad	0.79 ± 1.19d	1.11± 1.47ad	0.61± 1.11d
Height Z-score	0.61± 0.97d	0.23 ± 0.87d	0.48± 1.13d	0.13± 0.95d
Fat-free mass (kg)	25.3± 3.8d	44.3± 12.6bd	23.6± 4.8d	36.6± 6.4bd
Fat mass (kg)	8.6± 6.6d	12.3± 8.5bd	9.4 ± 6.9d	16.7± 8.5bd
Body fat percentage (%)	22.6± 11.5	20.9± 11.5bd	25.2± 11.1	29.8± 8.7bd
Phase angle 50 kHz (degrees)	4.95± 0.43bd	5.84± 1.02bd	4.68± 0.44bd	5.47± 0.76bd
IR Z 250 kHz/Z 5 kHz	0.791± 0.013ad	0.765 ± 0.028bd	0.797± 0.014ad	0.773± 0.021bd

mean±standard deviation

Statistical analysis by ANOVA and Tukey test for pairwise comparisons

Male vs female sex: a = *p* < 0.05; b = *p*< 0.01

Adolescents vs children: c = *p*<0.05; d = *p*<0.05

In line with body mass, both FFM (+ 75% in male and + 55% in female sex) and FM (+ 43% in males and + 78% in females) were higher in adolescents compared to children. The difference in %BF was observed only in the female sex (+ 18%).

As shown in Table 1, PhA was higher (Cohen’s *d* = 0.62) and IR lower (Cohen’s *d* = 0.44) in male than female participants both in the first and the second decade of life. It was also higher in adolescents compared to children within the same sex (Cohen’s *d* = 1.12 in male and 1.27 in female participants, when children vs adolescents were compared). Notably, PhA values were slightly but significantly higher, and IR lower, for the right body side in both age groups and sexes (Supplementary Table 1).

More detailed information about Phase angle at 50 kHz and IR at 250 kHz/5 kHz separated by sex and year of age has been shown in supplementary Tables 2 and 3. For a more intuitive understanding, percentiles for PhA and IR were calculated. For PhA, the 5th and the 95th percentile were: for male sex, 4.20/5.75 (≤ 10 yrs) and 4.28/7.63 (> 10 yrs); for female sex, 4.00/5.40 (≤ 10 yrs) and 4.30/6.79 (> 10 yrs). For IR, the 5th and the 95th percentile were: for male sex, 0.769/0.814 (≤ 10 yrs) and 0.715/0.809 (> 10 yrs); for female sex, 0.773/0.822 (≤ 10 yrs) and 0.736/0.806 (> 10 yrs). Table 2 shows that both PhA and IR were significantly related to age and to several anthropometric or body composition variables in both sexes, with the highest correlation (around 0.700) observed for FFM. No significant associations were found between PhA or IR and weight Z-score, height Z-score or BMI Z-score (data not shown). In multiple regression analysis, age and FFM emerged as major predictors (p < 0.001) of PhA (standardized beta for FFM = 0.633 for males and 0.242 for females respectively). The same pattern was observed for IR (standardized beta for FFM = −0.624 in males and −0.280 in females). These models explained 61% and 54% of the total variance for PhA, and 61% and 52% of the total variance for IR, respectively. Similar findings were obtained after excluding participants with obesity (data not shown).

**Table 2:** Non-parametric correlations between raw BIA variables and demographic, anthropometric and body composition variables

Raw BIA variables	Age	Height	Weight	BMI	FFM	FM	%BF
Boys							
Phase angle at 50 kHz	0.654**	0.638**	0.625**	0.377**	0.711**	0.138*	−0.193**
Impedance ratio 250 kHz/5 kHz	−0.679**	−0.665**	−0.654**	−0.402**	−0.725	−0.188**	0.146*
Girls							
Phase angle at 50 kHz	0.689**	0.616**	0.618**	0.450**	0.683**	0.469**	0.281**
Impedance ratio 250 kHz/5 kHz	−0.695**	−0.636**	−0.639**	−0.452**	−0.689**	−0.497**	−0.315**

*BMI *body mass index, *FFM *fat-free mass, *FM *fat mass, *%BF *percentage of body fat *p<0.05; **p<0.001

**Fig. 1:**
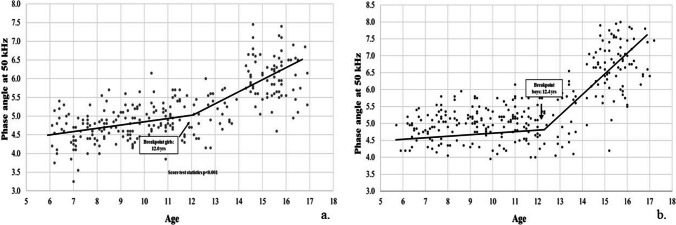
Trajectory of phase angle at 50 kHz in girls (panel a) and boys (panel b)

**Fig. 2:**
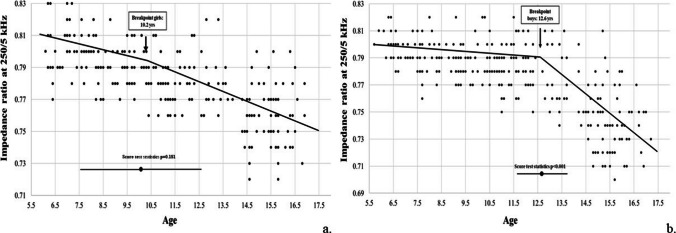
Trajectory of impedance ratio at 250 kHz/5 kHz in girls (panel a) and boys (panel b)

A linear relationship between PhA and age was observed from 5 to 10 yrs, which was similar in male and female participants (< 0.1 degrees/yr); a steeper slope was observed during adolescence, with a statistically significant breakpoint around 12 yrs in females and 12.4 yrs in males. After the breakpoint, the slope increased approximately tenfold in male and threefold in female adolescents (Table 3, Figs 1 and 2 and supplementary Figs. 1 and 2).

**Table 3:** Ontogenetic models of the impedance ratio and phase angle variables

	Slope First segment	Slope Second segment	Breakpoint (age in yrs)	Score test for the presence of a breakpoint
Boys				
Phase angle at 50 kHz	0.047 (0 to 0.093)	0.489 (0.394 to 0.584)	12.4 (11.7 to 13.1)	< 0.001
Impedance ratio 250 kHz/5 kHz	−0.002 (−0.004 to −0.001)	−0.012 (−0.015 to −0.009)	12.6 (11.7 to 13.5)	< 0.001
Girls				
Phase angle at 50 kHz	0.086 (0.039 to 0.134)	0.291 (0.217 to 0.366)	12.0 (10.7 to 13.4)	< 0.001
Impedance ratio 250 kHz/5 kHz	−0.003 (−0.006 to −0.001)	−0.006 (−0.007 to −0.004)	10.2 (6.9 to 13.5)	0.181

In contrast, IR decreases in both sexes during the first decade (around −0.003/yr). A significant breakpoint was detected at the age of 12.6 yrs in males –the breakpoint was not significant in female adolescents, with a sixfold steeper negative slope in the second compared to the first segment.

The trajectories did not substantially change after excluding participants with obesity (149 children, including 40 with severe obesity). A linear relationship between PhA or IR and age was still found in both sexes from 6 to 10 years, with breakpoints similar to those observed in the full sample (data not shown).

## Discussion

This study has examined sex- and age-related differences of BIA-derived raw variables (i.e., PhA and IR) in healthy Italian children and adolescents. It describes the trajectories of PhA and IR over the first two decades of life, showing biphasic relationships with age and strong relationships with FFM.

The assessment of body composition is helpful for evaluating nutritional status during growth and for monitoring the impact of diseases and nutritional interventions. As an example, fat mass index (i.e., body fat mass relative to height) could be considered for a better diagnosis of excess body fat and obesity [30]. Our findings align with previous studies using BIA [11, 31], which indicated that FFM and FM are greater in adolescents compared to children and that differences in %BF between sexes become evident during the second decade of life. It is worth noting that the BIA equation used to estimate body composition was originally developed in a US population, but indeed validated in children and adolescents from several ethnic backgrounds [27].

BIA-derived raw variables provide valuable, specific information on body composition: as a matter of fact, PhA and IR are considered proxy indexes of BCM, ICW/TBW ratio, and cellular integrity in both healthy and sick children/adolescents [13–18]. Thus, these two parameters may be used for evaluating muscle quality and health-related fitness and for monitoring nutritional status [18]. Given that a proper use of PhA and IR requires an understanding of their physiological variations, limited information is available on the changes of PhA over the first two decades of life [16], and no data have so far been provided for IR. In this study mean values of PhA or IR were used to retrieve information on both body sides. The same conclusions may be reached when considering the right or left side separately (data not shown).

As a general observation, our results show that sex-related differences in PhA were apparent in both the first and second decades of life. In contrast, previous studies showed that the difference was very small in children and became clear in adolescents [19]. Similarly, a difference in BIA vector between sexes was also observed at ages 14–15 yrs but not in younger adolescents and children [23].

In line with the available literature [1, 9, 13, 16, 19, 20, 23], PhA was also higher in adolescents compared to children (+ 17% in both sexes). Mean values are comparable with those reported by Schmidt et al. (2019) but slightly higher than the ones reported by Mattiello et al. [9, 21]. Consistent with previous findings [16], In addition, PhA was also found to be significantly associated with age and several anthropometric and body composition variables, especially FFM. No significant associations were found between PhA and weight or height Z-scores, and, in contrast to a recent study [9, 31], none were observed with BMI Z-score either.

The trajectory of PhA across the first two decades of life has not yet been described in a systematic way and sometimes illustrated only through figures [9, 20]. To fill the gap – it was the major objective of the study –, segmented regression and breakpoint analysis were performed. Given major changes in body composition during adolescence, distinct phases in the increase of PhA with age could be hypothesized. Actually, our findings showed two linear segments, with a breakpoint at around 12 yrs of age in both sexes (Table 3 and Figs. 1 and 2). Changes in PhA are likely due to the progressive increase in both BCM and the ICW/TBW ratio, as already indicated in previous studies [9, 31]. Indeed, the difference in slopes was more pronounced in male than female participants. This is consistent with findings showing similar PhA values up to age 13, with differences observed in the 14–15-year age group [9].

Although less commonly used, IR (determined with multi-frequency BIA) is also (inversely) related to BCM and ICW/TBW ratio. This is because at higher frequencies (e.g., 200 or 250 kHz) the alternating current penetrates cell membranes and flows through TBW, while at lower frequencies (e.g., 5 kHz) it mostly passes through ECW. Thus, because of the strong inverse relationship with PhA, IR might be used as a complementary parameter to PhA.

To our knowledge, this is the first study that provides consistent data on IR in the first decades of life. Our findings indicated that IR was significantly lower in adolescents than in children and in male compared to female adolescents and it was associated with FFM and age. In addition, it was inversely related to age, with a steeper slope during the second decade of life. Our findings do not allow us to establish why a more evident breakpoint was detected in male compared to female participants. Actually, further studies are needed to explore the usefulness of measuring IR in addition to PhA.

It should be noted that the trajectories of PhA and IR did not change after excluding participants with obesity, with breakpoints similar to those identified in the entire sample (data not shown). However, due to the small number of these children and adolescents, our data do not allow for specific conclusions in this subgroup.

Some other general remarks may also be of value. Given that hydration status can affect PhA and IR, the occurrence of severe hyperhydration or dehydration among participants in this study was considered improbable. A major effect of menstruation was also unlikely, as female adolescents were all evaluated in the early phase of the menstrual cycle. In the absence of pubertal staging, an association between breakpoints or differences in breakpoints and puberty remains suggestive but only speculative. As a tentative hypothesis, they could be due to variations in body composition that occur during sexual development, with a more pronounced increase in FFM (to which PhA is more strongly related). Previous studies have also reported the effect of sexual maturity on bioelectrical values and may help interpret the different breakpoints observed in the study; as example, sexual maturity has been associated with higher PhA values in adolescent girls [16, 32] and in female adolescent athletes [33].

In this study all anthropometric and body composition measurements were performed by trained operators under standardized conditions, and data were analysed using univariable and multivariable models. Indeed, some limitations should be acknowledged. As this is a single centre study (carried out in Southern Italy), the generalizability of the findings may be limited. Due to the small number of participants with obesity, a detailed analysis in this subgroup was not possible. The cross-sectional design did not allow to analyse the within-individual changes over time.

Body composition was assessed using BIA, a widely used and reliable technique, but no comparisons were made with reference methods (which indeed are challenging to implement). Finally, data on pubertal stage were not available, owing to the school setting.

In conclusion, BIA-derived raw variables significantly change over the first two decades of life, with a progressive increase in PhA and a decrease of IR. PhA or IR are strongly associated to FFM in both sexes. This study provides a comprehensive description of the trajectories of PhA and IR in healthy children and adolescents using segmented regression and breakpoint analysis. For both variables, breakpoints were observed around 12 yrs of age, followed by more marked variations with time. However, in the absence of pubertal staging, the association between these breakpoints and pubertal development remains probable and suggestive, and needs to be confirmed by further studies.

## Supplementary Information

Below is the link to the electronic supplementary material.

Supplementary Figure 1 - Trajectories of phase angle at 50 kHz in boys and girls. (JPG 106 KB)

Supplementary Figure 2 - Trajectories of impedance ratio at 250 kHz / 5 kHz in boys and girls. (JPG 101 KB)

Supplementary  Tables(DOCX 23.2 KB)

## Data Availability

No datasets were generated or analysed during the current study.
