# Supplementary figures and images for: The trajectories of bioelectrical impedance analysis-derived raw variables (phase angle and impedance ratio) in healthy Italian children and adolescents: a retrospective observational study
Source: Eur J Pediatr. 2026 Jan 24;185(2):103. doi: 10.1007/s00431-026-06748-2 (PMC12831691; doi:10.1007/s00431-026-06748-2)

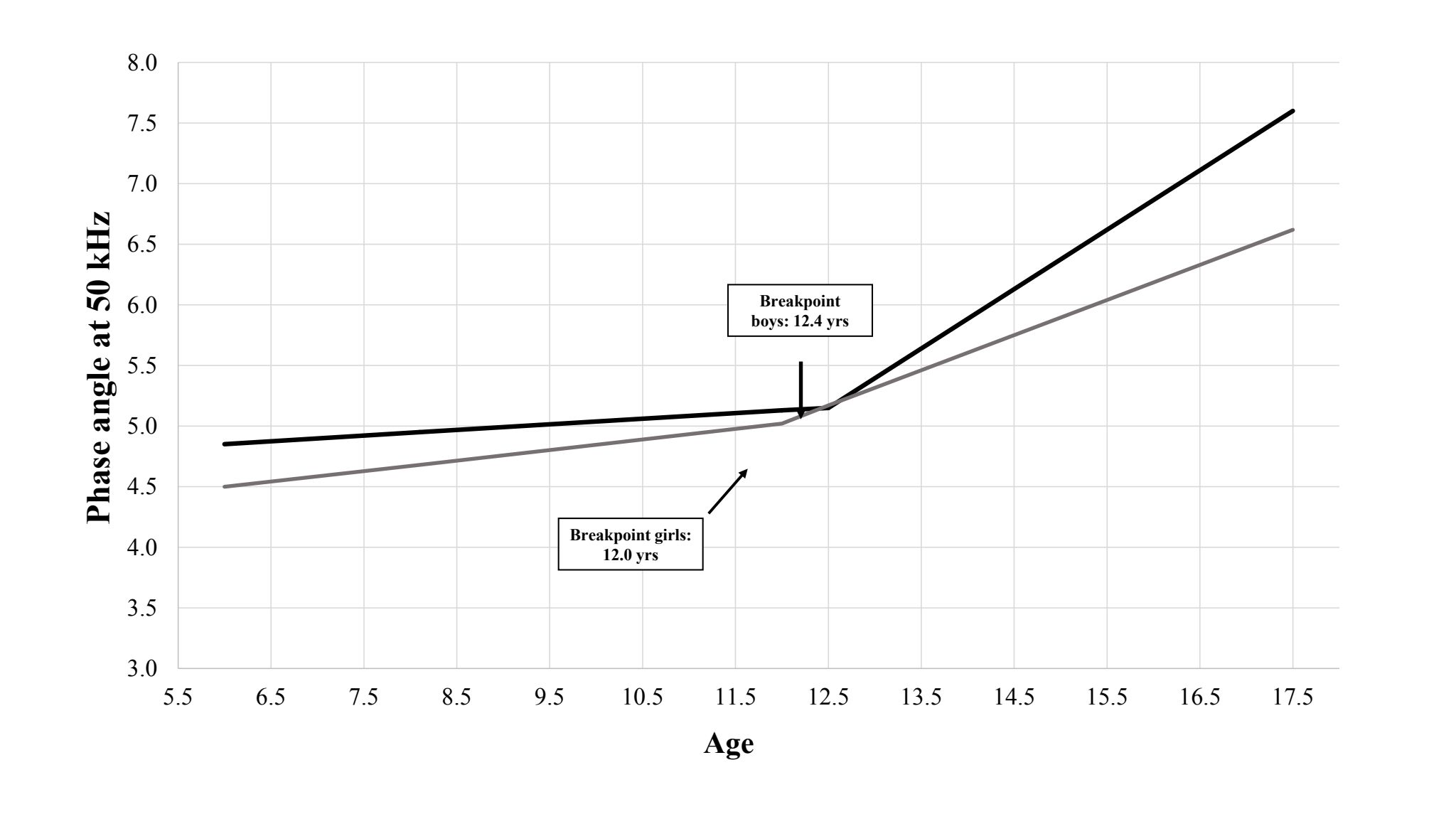

Supplement: Supplementary file 1 — Supplementary Figure 1 - Trajectories of phase angle at 50 kHz in boys and girls. (JPG 106 KB) [file 431_2026_6748_MOESM1_ESM.jpg]

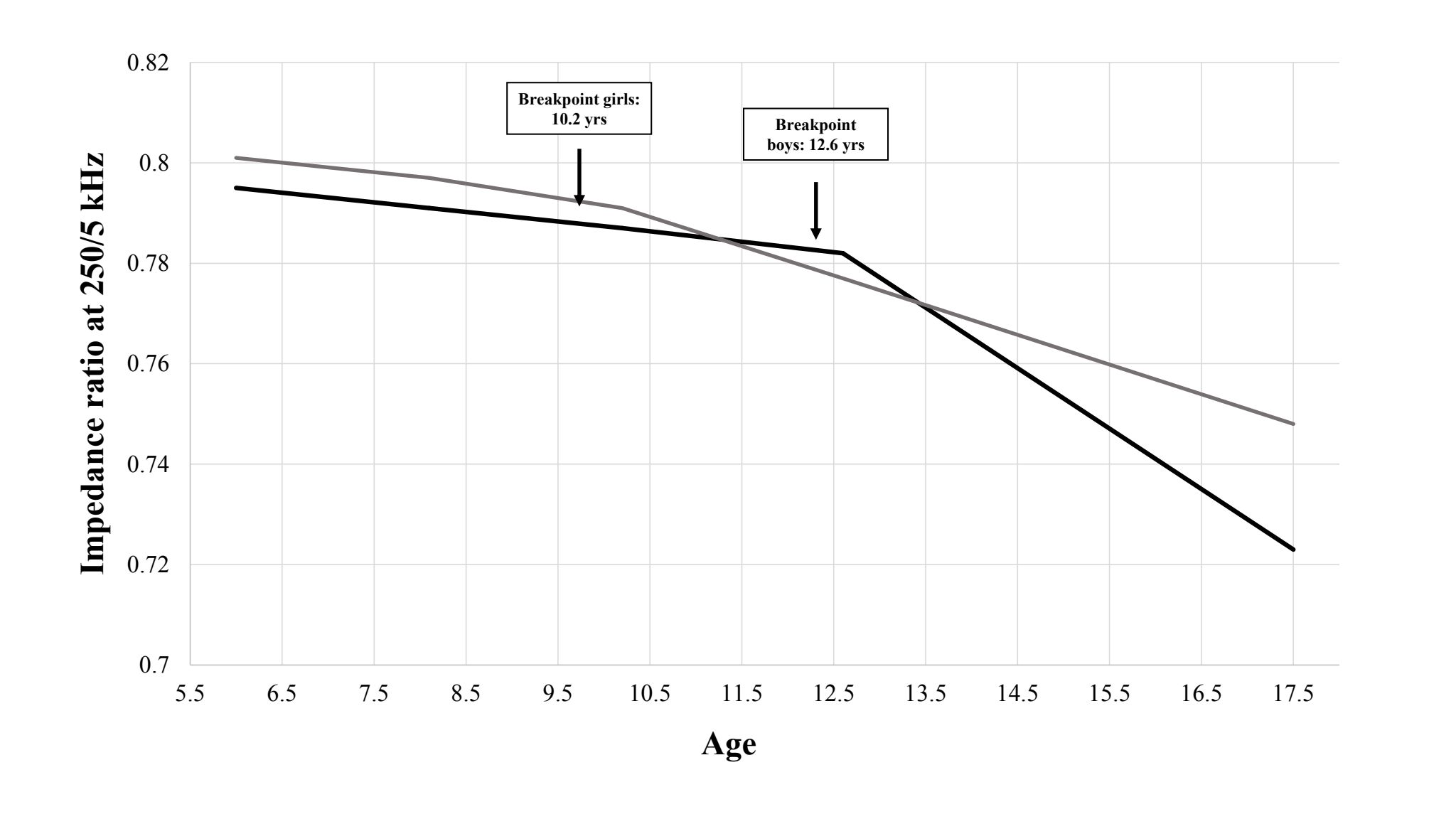

Supplement: Supplementary file 2 — Supplementary Figure 2 - Trajectories of impedance ratio at 250 kHz / 5 kHz in boys and girls. (JPG 101 KB) [file 431_2026_6748_MOESM2_ESM.jpg]
