# Supplementary material for: The trajectories of bioelectrical impedance analysis-derived raw variables (phase angle and impedance ratio) in healthy Italian children and adolescents: a retrospective observational study
Source: Eur J Pediatr. 2026 Jan 24;185(2):103. doi: 10.1007/s00431-026-06748-2 (PMC12831691; doi:10.1007/s00431-026-06748-2)
Supplement: Supplementary file 3 — Supplementary Tables(DOCX 23.2 KB) [file 431_2026_6748_MOESM3_ESM.docx]

| **Phase angle at 50 kHz in girls** | | | | | | | | | | | |
| --- | --- | --- | --- | --- | --- | --- | --- | --- | --- | --- | --- |
| **5** | **6** | **7** | **8** | **9** | **10** | **11** | **12** | **13** | **14** | **15** | **16** |
| n=0 | n=20 | n=31 | n=31 | n=32 | n=32 | n=26 | n=18 | n=12 | n=23 | n=36 | n=15 |
| - | 4.65±0.50 | 4.49±0.42 | 4.75±0.40 | 4.81±0.42 | 4.81±0.48 | 5.05±0.44 | 5.02±0.55 | 5.15±0.36 | 6.13±0.59 | 6.04±0.56 | 5.99±0.64 |
| **Phase angle at 50 kHz in boys** | | | | | | | | | | | |
| **5** | **6** | **7** | **8** | **9** | **10** | **11** | **12** | **13** | **14** | **15** | **16** |
| n=2 | n=22 | n=32 | n=21 | n=35 | n=29 | n=36 | n=23 | n=19 | n=22 | n=43 | n=18 |
| 4.58±0.53 | 4.84±0.36 | 4.95±0.41 | 5.00±0.52 | 5.03±0.42 | 5.01±0.46 | 5.07±0.54 | 5.11±0.60 | 5.43±0.71 | 6.58±0.72 | 6.70±0.79 | 6.90±0.71 |

**Supplementary table 1**. Mean and standard deviation of Phase angle at 50 kHz in children and adolescents separated by sex and year of age.

| **Impedance ratio at 250 kHz / 5 kHz in girls** | | | | | | | | | | | |
| --- | --- | --- | --- | --- | --- | --- | --- | --- | --- | --- | --- |
| **5** | **6** | **7** | **8** | **9** | **10** | **11** | **12** | **13** | **14** | **15** | **16** |
| n=0 | n=20 | n=31 | n=31 | n=32 | n=32 | n=26 | n=18 | n=12 | n=23 | n=36 | n=15 |
| - | 0.799±0.016 | 0.804±0.012 | 0.795±0.013 | 0.791±0.011 | 0.792±0.014 | 0.780±0.013 | 0.783±0.017 | 0.780±0.015 | 0.756±0.019 | 0.761±0.016 | 0.762±0.020 |
| **Impedance ratio at 250 kHz / 5 kHz in boys** | | | | | | | | | | | |
| **5** | **6** | **7** | **8** | **9** | **10** | **11** | **12** | **13** | **14** | **15** | **16** |
| n=2 | n=22 | n=32 | n=21 | n=35 | n=29 | n=36 | n=23 | n=19 | n=22 | n=43 | n=18 |
| 0.804±0.012 | 0.797±0.011 | 0.790±0.012 | 0.794±0.014 | 0.788±0.012 | 0.786±0.014 | 0.784±0.016 | 0.783±0.016 | 0.774±0.022 | 0.748±0.021 | 0.744±0.024 | 0.738±0.022 |

**Supplementary table 2**. Mean and standard deviation of Impedance ratio at 250 kHz / 50 kHz in children and adolescents separated by sex and year of age.

| N=580 | **Phase angle at 50kHz** | | | | **IR at 250 kHz /5 kHz** | | | |
| --- | --- | --- | --- | --- | --- | --- | --- | --- |
|  | Right side | | Left side | | Right side | | Left side | |
| **Boys** |  |  |  |  |  |  |  |  |
| Children (5-10 yrs) n=113 | 4.97 | ±0.44 | 4.93 | ±0.44a | 0.790 | ±0.013 | 0.792 | ±0.014a |
| Adolescents (11-17 yrs) n=189 | 5.90 | ±1.03 | 5.79 | ±1.02a | 0.764 | ±0.028 | 0.766 | ±0.028a |
| **Girls** |  |  |  |  |  |  |  |  |
| Children (5-10 yrs) n=115 | 4.71 | ±0.44 | 4.64 | ±0.45a | 0.796 | ±0.014 | 0.798 | ±0.014a |
| Adolescents (11-17 yrs) n=163 | 5.52 | ±0.77 | 5.42 | ±0.77a | 0.772 | ±0.020 | 0.775 | ±0.022a |

mean±standard deviation

a = p < 0.05 right side vs left side
